# Effects of roasting on kernel peroxide value, free fatty acid, fatty acid composition and crude protein content

**DOI:** 10.1371/journal.pone.0184279

**Published:** 2017-09-13

**Authors:** Shahla Hosseini Bai, Ian Darby, Tio Nevenimo, Godfrey Hannet, Dalsie Hannet, Matthew Poienou, Elektra Grant, Peter Brooks, David Walton, Bruce Randall, Helen M. Wallace

**Affiliations:** 1 Genecology, Faculty of Science, Health, Education and Engineering, University of the Sunshine Coast, Maroochydore DC, Queensland, Australia; 2 National Agricultural Research Institute, Islands Regional Centre – Keravat, Kokopo, East New Britain Province, Papua New Guinea; University of Illinois, UNITED STATES

## Abstract

Roasting nuts may alter their chemical composition leading to changes in their health benefits. However, the presence of testa may alleviate the negative effects of thermal treatments. Hence, this study aimed to explore the effects of roasting on kernel chemical quality and colour development of *Canarium indicum* and examine to what extent testa would protect kernels against damage from roasting. Roasting decreased peroxide value but increased free fatty acid, probably due to increased cell destruction and lack of enzyme inactivation, respectively. Protein content of kernels significantly decreased after roasting compared to raw kernels. However, testa-on kernels contained significantly higher protein content compared to testa-off kernels. Whilst colour development and mottling were observed in temperatures beyond 120°C, roasting did not alter fatty acid compositions of kernels. The mild roasting and presence of testa in kernels can be used to enhance health benefits of kernels.

## Introduction

Both growing population and global climate change bring challenges for food security [1–3]. Exploring new source of food in forests may provide a safety net when there is a food shortage [4]. Edible tropical nuts can be explored and commercialised to address food shortage in the world [5–7] because nuts are an important source of nutrients, unsaturated fats and proteins, and when consumed both roasted and raw have benefits for human health [8]. However, heat treatment and duration may alter texture, colour, and chemical content of the kernels including proteins and lipids [9–15]. Factors such as nut freshness, moisture content and post-harvest processing, including whether nuts are whole, ground, and testa-on or testa-off may influence kernel quality under different roasting conditions [16–19]. For example, testa-on almonds were less damaged by roasting compared to peeled almond due to having additional protection from the testa against oxidation under roasting conditions [16] and yet it has not been extensively studied. It is important that post-harvest processing of nuts does not negatively affect nut nutritional values to ensure their health benefits.

Roasting may alter peroxide value (PV) and free fatty acids (FFA) of nuts, which are indicative of nut rancidity. PV and FFA are evidence of autoxidation (free radical reaction) and hydrolytic rancidity, respectively [7, 10]. Hydro-peroxides produced from autoxidation react with other nut components (e.g. amino acids and proteins) leading to nut rancidity [7, 10]. The reaction of lipids with water is facilitated by different enzymes including esterases and lipases, is called hydrolytic rancidity producing FFA [20]. Acceptable FFA values vary for different nuts. For example, FFA of less than 1.5% and 1.0% are acceptable for walnuts and almonds, respectively [20]. Both PV and FFA can be influenced by roasting conditions but through different mechanisms [10, 13]. For example, PV is decomposed to highly unstable secondary oxidation products under heat, whereas FFA is influenced by heating due to decreased water content of the kernels and/or enzyme activity alteration [10, 21]. However, the changes of chemical content in kernels caused by roasting may differ in kernels collected from different species or even varieties of one species [10].

Nuts and vegetables are usually rich in unsaturated fats and are beneficial for human health [8]. Unsaturated fats decrease heart disease, diabetes and blood cholesterol [8]. Unsaturated fats however are more prone to rancidity than saturated fats when heated [13]. The oleic:linoleic (both unsaturated fats) ratio is also considered an important factor to assess nut quality and a decreased ratio indicates decreased oil oxidative stability [22, 23]. Consequently, it is important to explore to what extent roasting alters fatty acid composition of nuts.

*Canarium indicum* L. (Burseraceae) is an indigenous forest tree that occurs in East Indonesia, Papua New Guinea, the Solomon Islands and Vanuatu and is valued as a traditional food source [6]. Burseraceae contains 18 genera with over 700 species and many of these species have both economic and medicinal values [24]. The genus canarium comprises over 100 species [5]. The commercialization of nuts from *C*. *indicum* has commenced and the tree is also used as a valuable shade tree in cocoa plantations [25]. *C*. *indicum* nuts are nutritionally valuable for consumers as they are rich in oil (above 70%) and contain protein and vitamin E [17, 26]. *C*. *indicum* kernels are also traditionally consumed fresh, dried and roasted [6]. Roasting may alter colour or quality of the kernels affecting their marketability [12, 27]. Hence, this study aimed to (a) explore the effects of roasting on kernel PV, FFA and fatty acid composition as well as kernel colour development; (b) examine to what extent the presence of testa on kernels protects kernels during roasting and (c) determines what roasting conditions affect kernel colour and mottling.

## Materials and methods

### Sample collection and preparation

**Fig 1:**
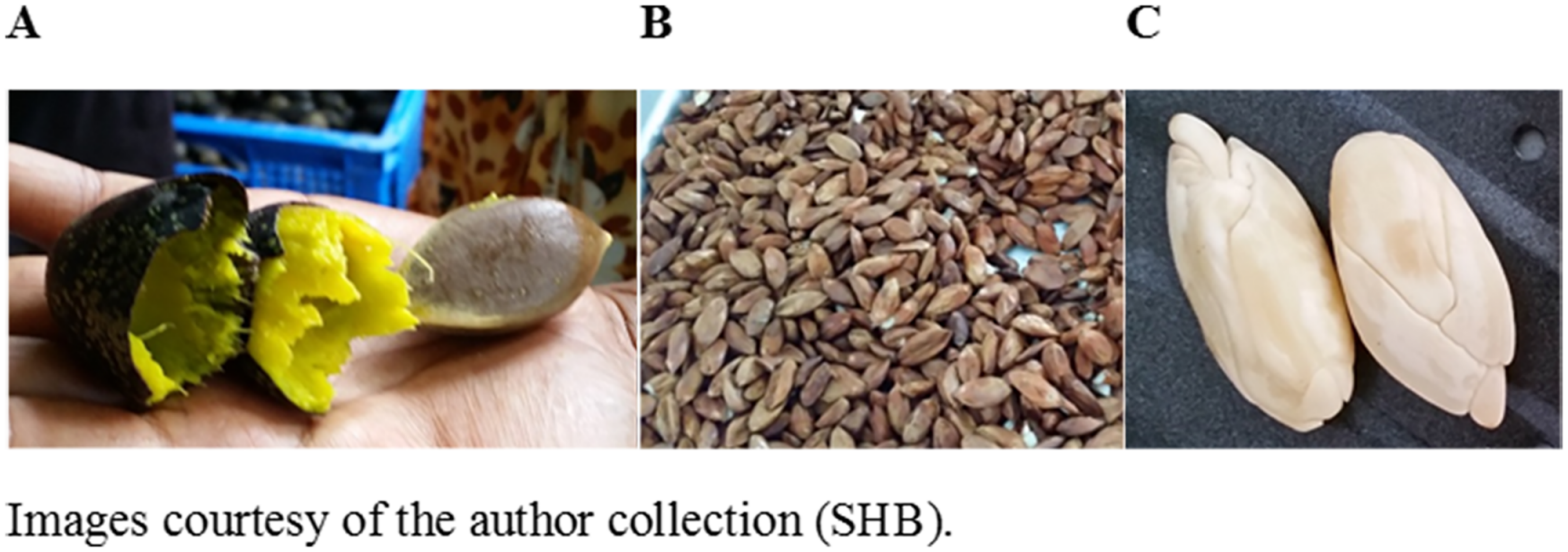
*Canarium indicum*—Nut and removed pulp (A), testa-on kernels (B) and testa-off kernels (C).

National Agriculture Research Institute (NARI) staff had purchased the purple fruits from different villages in Kerevat (4°21′S 152°2′E), East New Britain, Papua New Guinea (PNG) in December 2015. The fruits were mixed in the factory prior to sample collection for this study. It was impossible to identify landholders and therefore, no permission from private landholders was required. This study was permitted to be undertaken by NARI and did not involve endangered or protected species. NARI staff have also contributed in this research and are co-authored in this paper. The fruits were soaked in boiling water for 5 min to soften the pulp and the pulp was removed by squeezing (Fig 1A). After de-pulping, the nuts were dried and stored under ambient conditions at the *Canarium* processing factory located in NARI Kerevat, PNG for 12 weeks before further processing under ambient room temperature (on average 26.5°C) [7]. The nut in-shells are stored to be cracked when the nuts are out of season [7]. In February 2016, the shells were manually cracked and the kernels were blanched in hot water (100°C) for 90 seconds to soften the testa. The testa was then manually removed by squeezing [17, 28, 29]. The kernels were placed in zip-lock plastic bags and kept at 4°C before further analysis. A summary of kernel proximate chemical content has been presented in S1 and S2 Tables. In experiment 1, all the measurements taken from each treatment were replicated 5 times and 5 kernels were used for each replicate. All kernels were analysed within two weeks.

In experiment 2, another batch of purple *C*. *indicum* fruits was used to examine the effects of roasting on kernels with and without testa (called testa-on and testa-off, respectively) (Fig 1B and 1C). This batch of fruits was sampled in March 2016 and processed as described above. Since the time of fruit collection differed in this experiment compared to the roasting experiment, we have also provided a summary of kernel proximate chemical content for this batch of kernels in Supplementary Table 1. All measurements for each treatment were replicated 7 times and 5 kernels were used for each replicate. All kernels were analysed within two weeks.

**Table 1:** Probability of two-way ANOVA for oil volume, moisture content, peroxide value (PV), free fatty acid (FFA), fatty acid composition, total saturated fatty acid (TSFA), total unsaturated fatty acid (TUSFA), oleic:linoleic ratio and protein content of *Canarium indicum* after roasting.

	Oil volume	Moisture	PV	FFA	Palmitic acid	Stearic acid	Oleic acid (cis)	Oleic acid (trans)	Linoleic acid	TSFA	TUSFA	Oleic:linoleic	Protein
Temperature	**P<0.0001**	**0.011**	**P<0.0001**	**P<0.0001**	0.25	0.24	0.09	**0.005**	0.16	0.49	0.35	0.12	**P<0.0001**
Time	0.61	0.76	**P<0.0001**	0.12	0.87	0.34	0.49	0.79	0.17	0.51	0.30	0.27	**0.043**
Temperature × Time	0.64	0.17	**P<0.0001**	**0.002**	**0.052**	0.11	0.35	0.53	0.38	0.37	0.41	0.36	**0.006**
**Testa-on *vs* Testa-off**													
Temperature	0.111	ND	ND	**0.025**	**0.008**	**0.006**	0.424	0.513	0.839	0.232	0.232	0.11	0.823
Testa	0.811	ND	ND	**P<0.0001**	0.228	**0.019**	0.477	**0.012**	0.547	0.137	0.137	0.33	**P<0.0001**
Temperature × Testa	0.109	ND	ND	**0.036**	0.037	0.129	0.203	0.631	0.365	0.222	0.222	0.27	0.109

ND: Not determined.

### Roasting condition and experimental design

In experiment 1, the kernels were placed in aluminium trays and were air roasted in a laboratory oven, Memmert Gmbh and Co. KG, Schwabach, Germany. The roasting temperature were 110°C, 120°C and 150°C. The kernels were roasted for 5 min, 10 min and 20 min at each temperature. The kernels roasted at 150°C for 20 min turned dark brown which is not commercially acceptable and hence was not further analysed. The moisture content of kernels for each treatment was determined by placing kernels in oven at 105°C for 24 h [30]. In experiment 2 (testa-off *vs*. testa-on), testa-off and testa-on samples were roasted at two temperatures, 110°C and 120°C, for 10 min. The testa of the kernels were removed by hand before oil extraction and protein analyses.

### Oil extraction

The 5 kernels of each replicate (approximately 10 g) were crushed three times using a garlic crusher. The crushed kernels were added to 80 ml of pentane. The mixture was then stirred for 20 min and the pentane was removed from the oil using an air-tight vacuum rotator, (BÜCHI Labortechnik AG, Switzerland), for 15 min. The extracted oil was transferred to 5 ml glass vials and stored at 4°C before further analyses.

### Determination of peroxide values (PV) and free fatty acids (FFA)

The peroxide value (PV) of the oils was determined using a titration method according to AOAC Official Method 965.33 [31] with slight modification for micro-titration. In brief, 1 g of oil was placed into a 50 mL conical flask. Afterwards, 5 mL CH_3_COOH/CHCl_3_ was added to the oil and agitated gently to dissolve. Saturated KI solution (0.1 mL) was added followed by 1 min shaking and adding 6 mL deionised water to the mixture. The mixture was then slowly titrated using 0.01 *M* Na_2_S_2_0_3_. The PV expressed as milliequivalents of O_2_ kg^-1^ oil was calculated according to the following equation:

$$PV=\frac{\left(S-B\right)\times N\times 1000}{sample\;wt\;\left(g\right)\times \;1000}$$ (1)

Where:

*S* = sample titration (μL)

*B* = blank titration

*N* = normality of Na_2_S_2_O_3_

The FFA content (as *oleic*) of the crude oil was determined using a titration method described in AOAC method 940.28 [32] with a slight modification for micro-titration. Briefly, 1 g of oil was added to 7 mL ethanol previously neutralised to phenolphthalein. The titration was undertaken by adding 0.1 *M* NaOH to the mixture. The FFA was calculated using the following equation:

$$\%\;FFA\;\left(as\;oleic\right)=\frac{\left(\mu L\right)alkali\;\times \;N\;of\;alkali\;\times \;28.2\;mg}{sample\;wt\;\left(g\right)\;\times \;1000}$$ (2)

### Determination of protein and nutrient content of the kernels

Kernel total nitrogen (N) was obtained by combustion using a LECO TruSpec analyser. A conversion factor of 6.25 was used to convert the kernel N content to crude protein [33]. The nutrient content of the kernels were obtained on a Varian Vista Pro ICPOES instrument on samples open-vessel digested with a 5:1 mixture of nitric and perchloric acids as described for nutrient analysis of plant parts [34].

### Determination of fatty acid composition

To measure fatty acid composition, 1 μl oil was transferred into a 2 ml vial and 0.7 ml dry methanol solution containing butylated hydroxytoluene (BHT) and 25μl of 32% HCl were added to the vial. Afterwards, the mixture was incubated overnight (20 h) at 65°C. After incubation, 0.5 ml of hexane and 0.5 ml of MilliQ water were added and then the solution was shaken for 30 seconds followed by another wash with 0.5 ml of MilliQ water to further clean the oil. Afterwards, the upper layer was collected and was mixed with Na_2_SO_4_ to remove water from the oil in hexane. The fatty acid composition was then assessed using Gas Chromatography–Mass Spectrometry (GCMS) [35]. Total saturated fatty acid (TSFA) was a sum of palmitic acid and stearic acid; and total unsaturated fatty acid (TUSFA) was calculated as the sum of oleic acid (cis), oleic acid (trans) and linoleic acid.

### Kernel colour development

Kernels were assessed for colour development and mottling after roasting. As a standard for colour evaluation, a Taubmans Paints (Regents Park, Sydney, Australia) Colour Concepts^®^ colour swatch No.44 was employed. Kernels were ranked from 1 to 4 according to the four darkest colours on the swatch with 4 for the darkest colour: 1, pale; 2, lightly coloured; 3, moderately dark; 4, very dark. Mottled colour was ranked as 1 for slightly mottled, 2 for moderately mottled and 3 for severely mottled. Mottled colour was classified severe if approximately 20% or more of the surface examined had a mottled, brown appearance. Numbers of kernels in colour and mottled colour categories were calculated as percentages of total kernel number. The colour development was performed in temperatures of 110°C, 115°C, 120°C and 125°C at 10 min, 15 min and 20 min roasting time. At 150°C, we only examined 10 min roasting time.

### Statistical analysis

A two-way analysis of variance (ANOVA) was performed with temperature and roasting time as the main factors in Experiment 1. When interactions between temperature and exposure time was significant, a one-way analysis of variance (ANOVA) was performed to detect significant differences among each temperature and time combination followed by Tukey test where significant differences were observed. In the experiment 2, a two-way ANOVA was performed with roasting temperature and presence or absence of testa as main factors followed by a Tukey test. All data were tested for normality using Shapiro Wilk normality test and for homogeneity of variance using Levene's test. SPSS 21 software was used for all statistical analyses.

## Results

### The effects of different roasting conditions on kernel quality

**Fig 2:**
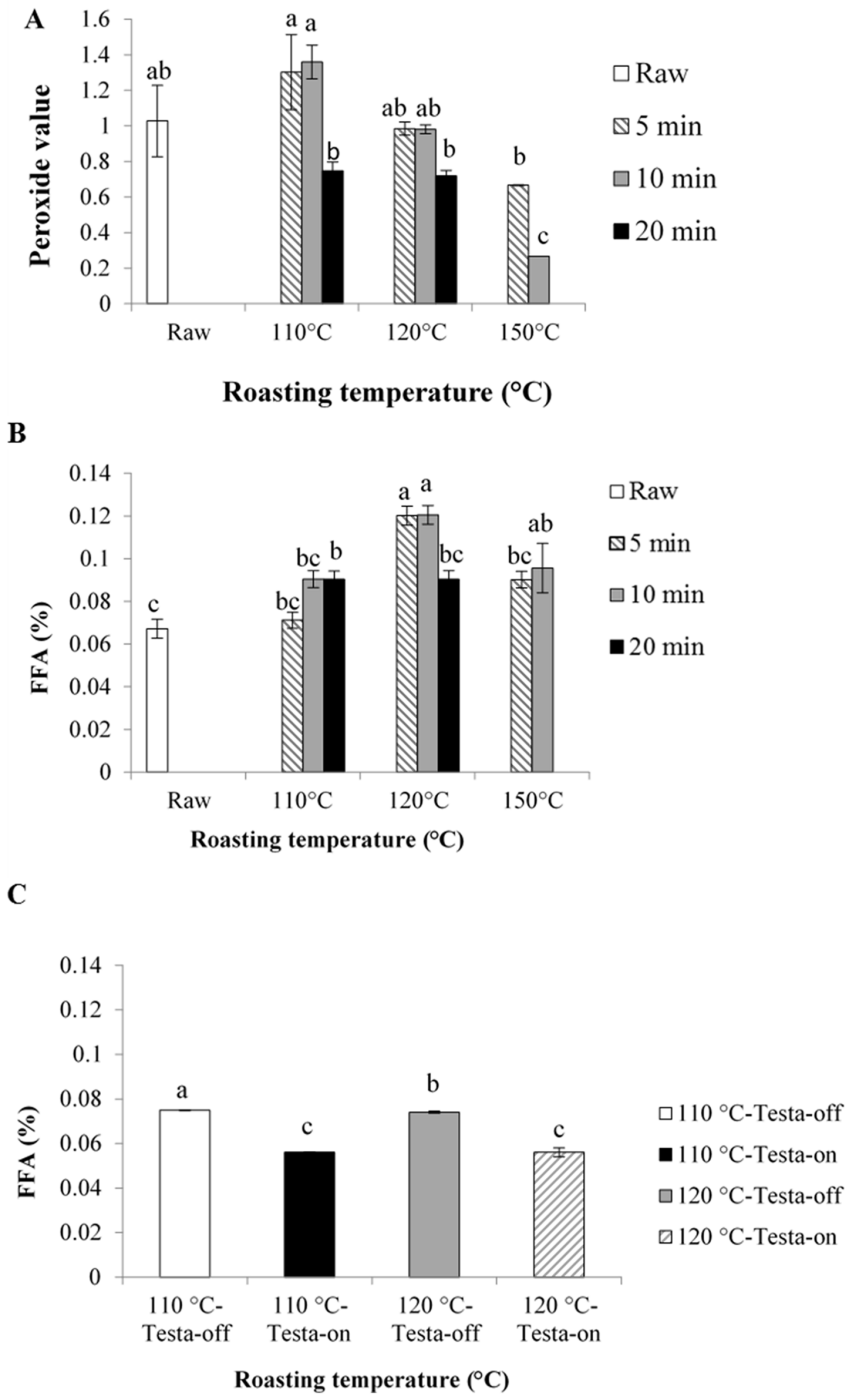
Peroxide values (A) and free fatty acid (FFA) (B) of the roasted *Canarium indicum* kernels at 110°C, 120°C and 150°C temperatures for 5 min (hatched column), 10 min (grey column) and 20 min (black column) compared to raw kernel (white column). FFA of the testa-off and testa-on kernels at 110°C (white and black columns, respectively) and at 120°C (grey and hatched columns, respectively) (C). Different lower case letters indicate significant differences at P<0.05.

The PV was influenced by both roasting temperature and exposure time (Table 1). Interaction between roasting temperature and exposure time was also significant (Table 1). Kernel PV significantly decreased only at 150°C for 10 min compared to other treatments including the raw kernels (Fig 2A). PV varied between 1.30 (millieq. O_2_ kg^-1^ oil) and 0.26 (millieq. O_2_ kg^-1^ oil) in this experiment.

The FFA was also influenced by roasting temperature and there was a significant interaction between roasting temperature and exposure time (Table 1). Roasting at 110°C at all exposure times did not change FFA compared to FFA of the raw kernels (Fig 2B). At 120°C, FFA was significantly greater in 120°C/5min and 120°C/10 min than that of all other treatments with an exception observed at 150°C/10 min (Fig 2B). At 150°C, FFA was significantly higher in 150°C/10 min that that of raw kernels but not at 150°C/5 min (Fig 2B).

**Fig 3:**
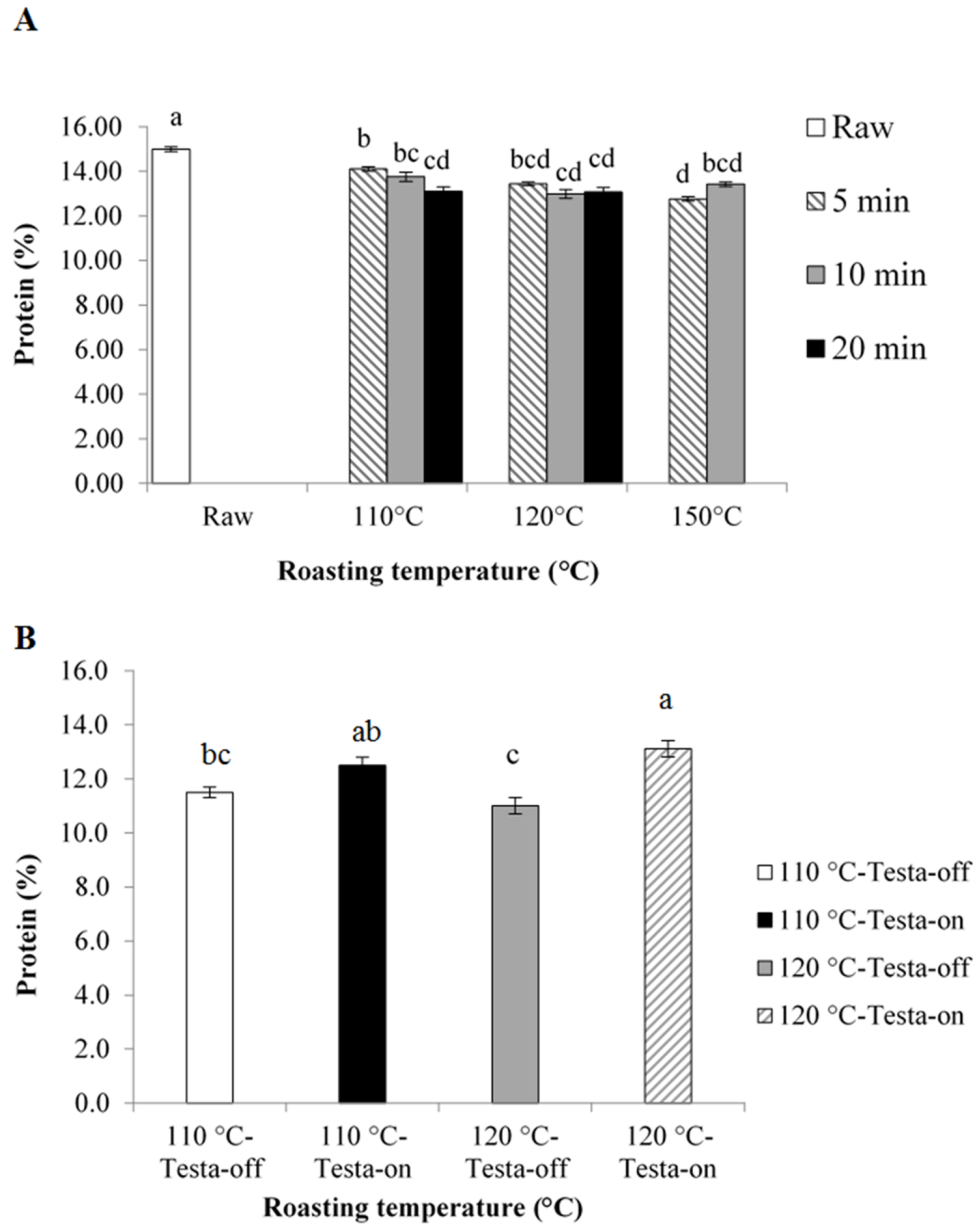
Protein content of the roasted *Canarium indicum* kernels at 110°C, 120°C and 150°C temperatures for 5 min (hatched column), 10 min (grey column) and 20 min (black column) compared to raw kernel (white column) (A). Protein content of the testa-off and testa-on kernels at 110°C (white and black columns, respectively) and at 120°C (grey and hatched columns, respectively) (B). Different lower case letters indicate significant differences at P<0.05.

Protein content of kernels was influenced by both roasting temperature and exposure time and there was a significant interaction between roasting temperature and exposure time (Table 1). Roasting significantly decreased protein content of kernels in all roasting conditions regardless of roasting temperature or duration of the exposure compared to raw kernels (Fig 3A). At 110°C, protein content of kernels roasted at 110°C/20 min was significantly lower than that of 110°C/5 min (Fig 3A). At both 120°C and 150°C, duration of exposure did not change protein content of the kernels (Fig 3A).

In general roasting did not affect fatty acid composition of the kernels with an exception observed in oleic acid trans where values were lower at 150°C for both 5 and 10 min (Tables 1 and 2). Oleic acid (*cis*) was the dominant fatty acid followed by palmitic acid and stearic acid (Table 2). The proportion of linoleic acid varied between 5.5% and 7.1% (Table 2). The detected oleic acid (*trans*) was the least of all fatty acids varied between 0.82% and 1.90% (Table 2). No influence of roasting was observed on the oleic:linoleic (O/L) ratio of the kernels. The O/L ratio of the kernels varied between 6.82 and 9.26 (Table 2).

**Table 2:** Fatty acid composition (percentages) of *Canarium indicum* under different roasting regimes. Paired bold cases represent a significant difference at P<0.05.

**Testa-off kernels under different roasting condition**									
	**Raw**	**110°C**			**120°C**			**150°C**	
		5 min	10 min	20 min	5 min	10 min	20 min	5 min	10 min
Palmitic acid	29.9(0.3)	29.3(0.3)	30.0(0.6)	29.1(0.6)	29.7(0.3)	28.2(0.5)	30.0(0.3)	28.9(0.6)	29.4(0.2)
Stearic acid	15.3(0.5)	14.8(0.3)	15.4(0.5)	14.6(0.5)	15.0(0.5)	16.9(0.6)	15.0(0.3)	15.8(0.7)	14.9(0.4)
Oleic acid (cis)	48.0(0.7)	49.0(1.2)	46.7(0.9)	49.7(0.9)	46.1(0.7)	47.2(1.2)	47.1(0.4)	48.0(0.3)	49.1(1.0)
Oleic acid (trans)	1.12(0.2)ab	0.83(0.3)ab	1.82(0.3)ab	0.90(0.3)ab	1.90(0.1)a	1.80(0.7)a	1.84(0.07)a	0.82(0.4)b	0.41(0.2)b
Linoleic acid	5.6(0.2)	5.8(0.7)	5.9(0.4)	5.5(0.4)	7.1(0.2)	5.7(0.2)	5.8(0.3)	6.3(0.2)	6.0(0.4)
TSFA	45.2(0.8)	44.2(0.4)	45.4(0.4)	43.8(0.5)	44.7(0.6)	45.2(0.5)	45.1(0.3)	44.8(0.3)	44.3(0.5)
TUSFA	54.7(0.8)	55.7(0.4)	54.5(0.4)	56.1(0.5)	55.2(0.6)	54.7(0.5)	54.8(0.3)	55.2(0.3)	55.6(0.5)
O/L	8.81(0.3)	8.99(0.9)	8.40(0.6)	9.26(0.4)	6.78(0.2)	6.82(1)	8.44(0.6)	7.82(0.3)	8.36(0.6)
**Testa-off *vs*. Testa-on kernels**									
		**110°C**			**120°C**				
		10 min	10 min		10 min	10 min			
		Testa-off	Testa-on		Testa-off	Testa-on			
Palmitic acid		31.1(0.5)	30.3(0.8)		27.0(0.9)	29.7(0.7)			
Stearic acid		**15.9(0.3)**	**17.5(0.3)**		**17.7(0.5)**	**18.2(0.3)**			
Oleic acid (cis)		45.0(1)	45.8(1.5)		48.0(1.5)	45.2(1)			
Oleic acid (trans)		**0.97(0.2)**	**0.23(0.1)**		**0.71(0.3)**	**0.19(0.1)**			
Linoleic acid		6.8(0.2)	5.9(0.6)		6.4(0.9)	6.6(0.6)			
TSFA		47.0(0.8)	47.9(1)		44.8(0.8)	47.9(0.7)			
TUSFA		52.9(0.8)	52.0(1)		55.1(0.8)	52.0(0.7)			
O/L		6.78(0.4)	8.16(0.9)		8.23(1.2)	7.15(0.7)			

TSFA–Total saturated fatty acid; TUSFA–Total un-saturated fatty acid; O/L–oleic:linoleic ratio

### Effects of roasting on testa-off and testa-on kernels

FFA was influenced by both roasting temperature and exposure time but no significant interaction between roasting temperature and exposure time was observed (Table 1). FFA of testa-on kernels was significantly lower than that of testa-off kernels at both 110°C and 120°C roasting temperatures (P<0.05; Fig 2C). Protein content of kernels was only influenced by the presence or absence of testa rather than roasting temperature (Table 1). Testa-on kernels contained higher protein content compared to testa-off kernels only at 120°C temperatures (P<0.05; Fig 3B).

Only stearic acid and oleic acid were influenced by the presence of the testa but in general the presence of testa did not alter TSFA, TUSFA and oleic:linoleic ratio compared to those without testa (Tables 1 and 2). In the testa-off *vs*. testa-on roasting trial, stearic acid was significantly higher in testa-on kernels than those of testa-off kernels (Table 2). In contrast, oleic acid (trans) significantly decreased in testa-on kernels compared to those of testa-off kernels (Table 2).

### Effects of roasting on kernel colour development

Dark kernels (rank 4) were not found until temperatures of 125°C were used (Table 3). All dark kernels in the experiment were found in the treatments roasted at 125°C. Dark kernels were few (from 3% to 6%). Severe mottled colour occurred in all the roasting treatments, but did not begin to increase markedly until the 120°C treatments, when it rose from 11% of the 10 minute treatment to 31% of the 15 minute treatment (Table 3).

**Table 3:** *Canarium indicum* kernels (%) in each colour category and mottled colour category for different roasting regimes.

Treatment	Colour_1	Colour_2	Colour_3	Colour_4	Mottling_1	Mottling_2	Mottling_3
110°C/10 min	60.83	38.83	-	-	69.00	27.67	3.33
110°C/15 min	69.17	30.50	-	-	55.33	38.83	5.84
110°C/20 min	44.17	49.67	-	-	49.83	38.67	11.50
115°C/10 min	27.50	71.83	-	-	38.67	55.33	6.00
115°C/15 min	5.50	77.33	16.67	-	25.00	63.50	11.50
115°C/20 min	44.17	55.33	-	-	72.00	27.83	0.17
120°C/10 min	16.50	63.50	19.33	-	24.83	63.50	11.67
120°C/15 min	-	68.83	30.33	-	-	69.00	31.00
120°C/20 min	-	58.17	41.50	-	-	74.50	25.50
125°C/10 min	-	47.00	49.83	3.17	5.67	66.33	28.00
125°C/15 min	-	47.00	49.67	3.33	-	58.00	42.00
125°C/ 20 min	-	30.50	63.50	6.00	-	47.17	53.83
150°C/ 10 min	2.77	27.77	63.88	5.55	5.55	72.22	22.23

## Discussion

Roasting decreased kernel PV and crude protein contents but slightly increased FFA. The presence of the testa reduced the effects of roasting on FFA and protein in kernels when compared to those without testa. There were no significant differences between raw and roasted kernels in fatty acid composition of the kernels.

The PV decreased significantly compared to raw kernels when roasting temperature reached 150°C with exposure duration of 10 min. Increased PV after roasting has been reported in walnuts [36]. A decrease of water activity due to roasting would lead to increased oxidation [37]. However, peroxides are not stable during heating and may start decomposing when the temperature increases [10]. Hence, decreased PV reported after roasting is in fact associated with its further conversion to carbonyl compounds with low molecular mass [38]. Hence, reported decreased PV after roasting does not indicate a lower PV production compared to not-roasted kernels but it may indicate a greater PV break down to secondary oxidation compounds [10, 21, 39].

In our experiment FFA increased after roasting with the highest FFA recorded at 120°C. Increased or decreased FFA after roasting has been observed in other nuts and attributed to hydrolytic enzyme inactivation, deterioration or no effect [10, 36]. Heat may reduce the activity of some enzymes involved in hydrolytic rancidity but some enzymes (e.g. esterase) remain active after heating leading to increased FFA values of the nuts [40]. Lipase activity may also be reduced in temperatures higher than 60°C but still could be detected [41]. It has been shown that lipase inactivation may require high temperature and long exposure time (e.g. 140°C for 42 min and 31 min in hazelnut and almond respectively) [42]. Nut cells and structures are also physically damaged after roasting as shown by electron microscopy technique [43]. Hence, damaged cells in the presence of hydrolytic enzymes can contribute to increased FFA after roasting. Although our study does not indicate what enzymes remained active after roasting leading to increased FFA, it suggests that some of the enzymes found in *C*. *indicum* kernels are heat resistant and remain active even at 150°C/10 min.

Our results showed that roasting *C*. *indicum* kernels in-testa may slow down development of kernel rancidity components, which is consistent with studies that revealed testa provides additional protection for kernels against heating and slowing down the rancidity processes [16, 38]. In our experiment, testa-on kernels contained lower FFA than those of the testa- off kernels. Increased FFA after roasting could be attributed to heat resistance of the enzymes as well as tissue disruption [36]. In our experiment, the testa may have decreased damage to the cells of the kernels leading to lower FFA production compared to testa-off kernels.

In our study roasting decreased protein content of roasted kernels compared to raw kernels, as indicated by reduced nitrogen content. Our finding is consistent with Özdemir et al., (2001) [10] who found nitrogen content of hazelnuts decreased with increased roasting temperature. Decreased nitrogen content after thermal treatments might be associated with increased amino acid concentration per unit of nitrogen due to loss of amides and amines [44]. Additionally, thermal treatments may generate amino acids in food [45] which further increases the concentration of amino acids per unit of nitrogen. We did not determine the amino acid changes in the *C*. *indicum* kernels. However, our results indicated that testa-on samples at higher temperatures retained higher nitrogen, probably due to an additional protection against heat when kernels were roasted at 110°C and 120°C.

Roasting regimes used did not alter fatty acid composition of the testa-off kernels. Our results were consistent with roasting studies on almond and hazelnut where no or negligible effects of roasting were observed on fatty acid composition [11, 22, 46]. In contrast, hazelnut roasting resulted in alteration of fatty acid composition with decreased linoleic acid and increased oleic and saturated fatty acids after roasting [13]. The alteration of fatty acid composition may occur at elevated temperatures. For example, fatty acid composition in hazelnut was altered when roasting at 165°C and 185°C with 15 min exposure time [13]. However, hazelnut was also roasted at 180°C for 21 min without any effect on fatty acid composition [11]. While it is difficult to explain these contradictory findings, we could suggest that the highest temperature (150°C) used in our experiment was not high enough to alter fatty acid composition.

In our experiment, oil oxidative stability was also not influenced by roasting conditions since oleic:linoleic ratio was not altered by roasting. Oleic acid of *C*. *indicum* was comparable to macadamia (51%) [47]. Linoleic acid content of *C*. *indicum* (5.5%–7%) was lower than pistachio (14%) and cashew (17%) but higher than that of macadamia (1.3%-2.4%) but was comparable to cashew and hazelnuts (approximately 7.5%) [47–49]. Both oleic and linoleic acids are unsaturated fats which are prone to autoxidation and hence are not heat resistant [38]. Hence, it is important that roasting does not alter unsaturated fats. Both oleic and linoleic acids are also important for health [50]. For example, oleic acid, also abundant in olive oil, contains phenols and has been shown to contribute many health benefits [48]. Linoleic acid is essential for health but nuts are not rich in linoleic acid with the exception of walnut [48]. Hence, the roasted nuts in this experiment were of similar fatty acid composition to raw kernels, which may suggest both roasted and raw kernels may have the same health benefits when consumed.

A greater colour development and mottling commenced in the temperatures beyond 120°C. Colour development under roasting can be associated with kernel quality [27, 43, 51]. Usually very dark and brown kernels are unacceptable [51]. Nonetheless, in the current trial, the kernel colour was not brown at the highest temperature. Hence, the kernel colour developed under roasting at 150°C should not affect the marketability of the kernels.

## Conclusion

This study evaluated the changes of chemical composition and oxidative stability of *C*. *indicum* kernels under different roasting regimes. Roasting regimes decreased kernel PV and increased FFA content which may have implication for the shelf-life of the roasted kernels. Roasting in general decreased protein content of the kernels but did not alter fatty acid composition of the kernels even at the highest temperature (150°C). The oxidative stability of the oil was also not influenced by roasting in this experiment as shown by an unchanged ratio of oleic:linoleic. Roasting also did not alter fatty acid composition of the kernels which may suggest both roasted and raw kernels have beneficial health effects. The testa provided additional protection against thermal treatments. Currently, the Galip (*C*. *indicum*) factory at NARI roasts the kernels at 150°C for 10 min and our study suggests that this roasting regime would not affect kernel chemical composition and oil oxidative stability nor excessive colour development affecting its marketability.

## Supporting information

S1 Table Nutritional composition of raw *Canrium indicum* kernel characteristics. Experiment roasting 1 represents kernels used for roasting temperate and duration of exposure. Experiment roasting 2 represents kernels used in testa-on and testa-off experiments.
(DOCX)

S2 Table Moisture content of the kernels after roasting used for Experiment roasting 1. Different lower case letters indicate significances at P<0.05.
(DOCX)
