# Supplementary material for: Effects of roasting on kernel peroxide value, free fatty acid, fatty acid composition and crude protein content
Source: PLoS One. 2017 Sep 13;12(9):e0184279. doi: 10.1371/journal.pone.0184279 (PMC5597184; doi:10.1371/journal.pone.0184279)
Supplement: S1 Table — Experiment roasting 1 represents kernels used for roasting temperate and duration of exposure. Experiment roasting 2 represents kernels used in testa-on and testa-off experiments. (DOCX) [file pone.0184279.s001.docx]

**S1 Table.** Nutritional composition of raw *Canrium indicum* kernel characteristics. Experiment roasting 1 represents kernels used for roasting temperate and duration of exposure. Experiment roasting 2 represents kernels used in testa-on and testa-off experiments.

|  | | | | |  |  |  |
| --- | --- | --- | --- | --- | --- | --- | --- |
|  | **Experiment**  **Roasting 1** | | **Experiment**  **Roasting 2**  **(Testa-on *vs* testa off)** | |  |  |  |
| Nitrogen (%) | 2.40 | (0.02) | 1.85 | (0.04) | |  |  |
| Crude protein (%) | 14.9 | (0.1) | 11.5 | (0.2) | |  |  |
| Calcium (mg kg^-1^) | 392 | (28) | 455 | (66) | |  |  |
| Copper (mg kg^-1^) | 9.90 | (0.3) | 9.43 | (0.7) | |  |  |
| Iron (mg kg^-1^) | 37.6 | (2) | 47.9 | (3.0) | |  |  |
| Potassium (%) | 0.61 | (0.02) | 0.62 | (0.01) | |  |  |
| Magnesium (%) | 0.28 | (0.01) | 0.39 | (0.01) | |  |  |
| Sodium (mg kg^-1^) | 6.29 | (1.1) | 17.5 | (2.7) | |  |  |
| Phosphorus (%) | 0.82 | (0.01) | 0.79 | (0.02) | |  |  |
| Sulphur (%) | 0.14 | (0.005) | 0.15 | (0.006) | |  |  |
| Zinc (mg kg^-1^) | 22.6 | (1.4) | 30.8 | (1.4) | |  |  |
| All data presented based on fresh weight. | | | | | | |  |
