# Supplementary material for: Effects of roasting on kernel peroxide value, free fatty acid, fatty acid composition and crude protein content
Source: PLoS One. 2017 Sep 13;12(9):e0184279. doi: 10.1371/journal.pone.0184279 (PMC5597184; doi:10.1371/journal.pone.0184279)
Supplement: S2 Table — Different lower case letters indicate significances at P<0.05. (DOCX) [file pone.0184279.s002.docx]

**S2 Table**. Moisture content of the kernels after roasting used for Experiment roasting 1. Different lower case letters indicate significances at P<0.05.

|  |  | Moisture content  (%) | |
| --- | --- | --- | --- |
| Raw |  | 4.09 | (0.9)a |
| 110 °C/5 min | | 2.14 | (0.01)ab |
| 110 °C/10 min | | 2.08 | (0.1)ab |
| 110 °C/20 min | | 0.96 | (0.2)ab |
| 120 °C/5 min | | 2.44 | (0.3)ab |
| 120 °C/10 min | | 2.01 | (0.07)ab |
| 120 °C/20 min | | 1.68 | (0.09)ab |
| 150 °C/5 min | | 1.86 | (0.03)b |
| 150 °C/10 min | | 1.14 | (0.08)b |
